# A Novel Equivalent Method for Computing Mechanical Properties of Random and Ordered Hyperelastic Cellular Materials

**DOI:** 10.3390/ma16216990

**Published:** 2023-10-31

**Authors:** Jian Li, Jianfeng Zhao, Qianhua Kan, Yuyu Tian, Li Yu, Yunqiang Peng, Xicheng Huang

**Affiliations:** 1 Shock and Vibration of Engineering Materials and Structures Key Laboratory of Sichuan Province, Mianyang 621999, China; 2 School of Mechanics and Aerospace Engineering, Southwest Jiaotong University, Chengdu 611756, China

**Keywords:** cellular materials, hyper-elasticity, equivalent calculation method, finite-element simulation, computational efficiency

## Abstract

Simulating the mechanical behavior of cellular materials stands as a pivotal step in their practical application. Nonetheless, the substantial multitude of unit cells within these materials necessitates a considerable finite element mesh, thereby leading to elevated computational expenses and requisites for formidable computer configurations. In order to surmount this predicament, a novel and straightforward equivalent calculation method is proposed for the computation of mechanical properties concerning both random and ordered hyper-elastic cellular materials. By amalgamating the classical finite element approach with the distribution attributes of cells, the proposed equivalent calculation method adeptly captures the deformation modes and force-displacement responses exhibited by cell materials under tensile and shear loads, as predicted through direct numerical simulation. This approach reflects the deformation characteristics induced by micro-unit cells, elucidates an equivalent principle bridging cellular materials and equivalent materials, and substantially curtails exhaustive computational burdens. Ultimately, this method furnishes an equivalent computational strategy tailored for the engineering applications of cellular materials.

## 1. Introduction

Cellular materials exhibit microstructure-controlled mechanical properties, behaving as structures at a small scale and as materials at a macroscopic scale [1]. They are widely studied and applied due to their advantages [2,3,4,5], i.e., lightweight, high specific strength, good thermal and acoustic insulators, a wide range of material mechanical properties, multifunction, etc. Originally stemming from foam or porous materials with random unit cells, they have evolved into honeycomb, lattice, architected cellular, and metamaterial forms with ordered unit cells. In practical applications, numerical simulation of cellular materials is the footstone of structural design [6,7,8].

Researchers have recently conducted many numerical simulations on cellular materials, which can be classified into three distinct categories:

(1) Equivalent constitutive model [9,10,11]. The unit cells of cellular materials can exhibit random or ordered arrangements. A brief simulation method was established according to the constitutive model for the continuous materials. When the macroscopic scale of cellular materials is much larger than the microscopic scale of their unit cells, they can be treated as continuous solid materials. Based on the experimental results, an equivalent constitutive model is developed to describe the macroscopic equivalent stress-strain curves. However, this method neglects the micro-architecture properties.
(2) Modeling based on representative volume element (RVE) [12,13,14,15,16,17,18,19,20]. Cellular materials include materials with either uniform or non-uniform unit cells. In the case of non-uniform cellular material, an RVE is constituted by enough characteristic unit cells or mesoscopic models [12,13,14]. Then, the simplified RVE, such as the primitive cubic model, Kelvin model, Voronoi model, and so on, is established. In the case of uniform cellular material [16,17], such as periodic lattices and triply periodic minimal surfaces, the RVE consists of specific unit cells. This method effectively describes the deformation behavior of cellular materials and reflects their basic structure characteristics (the micro-architecture shape), while also demonstrating computational efficiency. However, this method overlooks the influence of the micro-architecture parameters (size, wall thickness, distribution, and material properties) on the mechanical properties, thus emphasizing that selecting an appropriate RVE is crucial for accurately determining the overall mechanical properties.
(3) Modeling based on reconstructed microstructure [21,22,23,24,25]. With the development of computational ability and micro-computerized tomography, reconstructed microstructures can be used to simulate the deformation behavior of actual materials. The discrepancies arising from design and manufacturing contribute to the unstable mechanical responses observed in cellular materials through foaming or 3D printing [22,23,24]. By employing reconstruction technology, the actual micro-structural models are constructed for finite element simulation, enabling a comprehensive understanding of complex cellular materials and an accurate prediction of their deformation behaviors. However, this direct modeling method requires significant computational resources due to the incorporation of complex micro-architectures with large finite element meshes into finite element software, thereby limiting its practical applicability in engineering contexts.

In summary, ongoing research is focused on simulating the mechanical behavior of cellular material. This study endeavors to develop a novel and straightforward equivalent calculation method for predicting the mechanical properties of random and ordered hyper-elastic cellular materials. The developed method aims to accurately capture the characteristics of micro-architecture, enhance computational efficiency, and facilitate engineering applications. The organizational structure of this paper unfolds as follows: Section 2 presents the generic strategy of the equivalent calculation method. Subsequently, Section 3 delineates the detailed simulated procedure. Moving forward, Section 4 showcases the calculated results using this equivalent calculation method, along with discussions. Finally, Section 5 presents the conclusions.

## 2. Generic Strategy for Equivalent Calculation Method

**Figure 1:**
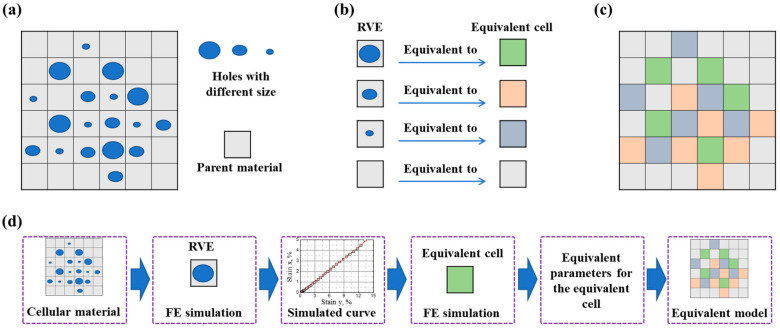
The schematic diagram of the generic strategy for the equivalent computational method: (**a**) cellular material with different pore sizes; (**b**) RVEs transforming to equivalent cells with different material parameters; (**c**) the equivalent model corresponding to (**a**); (**d**) the procedural essence of the equivalent computational method.

In this section, based on the equivalent principle of the stress–strain curve equivalence between cellular material and their corresponding equivalent material, we propose a novel and straightforward method for calculating the equivalent properties of cellular materials. This approach treats the cellular material as analogous to a continuous medium and employs a generic strategy depicted in Figure 1. Firstly, Figure 1a illustrates a cellular material with varying pore sizes. Subsequently, the cellular material is divided into multiple parts according to their structural features. These parts are classified and defined as various representative volume elements (RVEs), as illustrated in Figure 1b. These RVEs are further represented by uniformly equivalent cells with various material parameters to transfer their mechanical properties. Consequently, an equivalent model consisting of several equivalent cells is constructed to represent the original cellular material in Figure 1a, as shown in Figure 1c. Finally, the mechanical properties of cellular materials can be simulated using the equivalent model while ensuring the equivalence between the computed results in Figure 1c and those in Figure 1a. The procedural essence of this equivalent computational method is briefly summarized in Figure 1d.

Within this methodology, the cellular material under consideration can exhibit either a random or ordered nature. For random cellular materials with different micro-characteristics, the selection of RVEs aligns with the size and distribution of the micro-architecture, which can be derived from micro-computerized tomography. It is worth noting that the precision of the computational result increases as more RVEs are chosen. Thus, selecting an appropriate number of RVEs requires a delicate balance between accuracy and efficiency. This is the same as in the traditional approaches, where the number of elements can be reduced suitably without significantly reducing the accuracy of the evaluation. For the ordered cellular materials with regular unit cells (both uniform and non-uniform distributions), these unit cells themselves can serve as RVEs. Subsequently, the equivalent cells are implanted into the equivalent model based on the distribution of RVE. For simplicity, this study employs cellular materials with regular unit cells as illustrative examples to explore the potential of the equivalent calculation method.

## 3. Simulated Procedure

### 3.1. Simulated Model Generation

**Figure 2:**
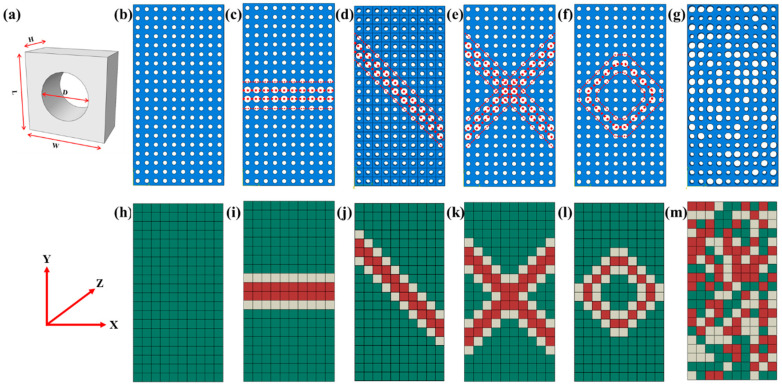
The cellular materials and their corresponding equivalent materials: (**a**) A plate unit cell with a circular hole in the thickness direction; (**b**) A plate with uniformly distributed cellular; (**c**) A cellular plate with stretching feature; (**d**) A cellular plate with shearing feature; (**e**) A cellular plate with double shearing feature; (**f**) A cellular plate with circular deforming feature; (**g**) A cellular plate with random deforming feature. (**h**–**m**) are the corresponding equivalent materials of cellular materials from (**b**–**g**). The green, white, and red codes denote the equivalent cells with pore diameters of 0.4, 0.6, and 0.8 mm, respectively.

The cellular materials in this study are based on polydimethylsiloxane (PDMS) with hyper-elastic properties as the basic material. To investigate the equivalent method mentioned in Section 2, we have established models of ordered and random cellular materials along with their corresponding equivalent material, shown in Figure 2. In this study, the cellular materials consist of rectangle unit cells measuring 1 × 1 × 1 mm^3^, with pore diameters of 0.8, 0.6, and 0.4 mm, as shown in Figure 2a. Based on the different topologies, cellular materials with dimensions of 10 × 20 × 1 mm^3^ are established to exhibit uniform deformation features in Figure 2b, stretching features in Figure 2c, shearing features in Figure 2d, double shearing features in Figure 2e, circular deforming features in Figure 2f, and random deformation features in Figure 2g. The models of the corresponding equivalent materials are depicted in Figure 2h–m. The plate with uniformly distributed cells will be used to validate the reasonability of the equivalent method and supply basic material parameters for simulating equivalent materials. The plate with different deformation features will be employed to verify the applicability of the equivalent calculation method and clarify the equivalent principles. The plate with randomly distributed cells will be used to predict the mechanical properties of the random cellular materials.

Two types of simulated model generation methods are employed, differing for the ordered and random models. (1) For the ordered cellular material: The cellular materials are first established by assembling the unit cells with varying pore diameters in the ABAQUS to form the cellular materials exhibiting different deformation features. Subsequently, continuum cubes measuring 10 × 20 × 1 mm^3^ are established and meshed with an element size of 1 mm. Material parameters corresponding to the distributions of cells with different pore diameters in the cellular materials are then assigned to cubic elements at their respective locations, resulting in computed models representing ordered equivalent materials. The determination of material parameters will be discussed in Section 3.3. (2) For the random cellular material, the randomness of the equivalent materials is achieved using the Python script. Firstly, the continuum cubes with dimensions of 10 × 20 × 1 mm^3^ are established. These cubes are then meshed with an element size of 1 mm to create a finite element model of random equivalent material. The combination of finite element software and Python (3.11) script is utilized for this purpose, and further details can be found in Appendix A. Subsequently, cellular materials are established by assembling the unit cells with different pore diameters based on the material parameter distribution of the random model. Finally, a finite element model of cellular materials with varying sizes of unit cells (as shown in Figure 2g) is constructed using the random information in Figure 2m.

### 3.2. Finite Element Modeling, Boundary Conditions, and Mesh

**Figure 3:**
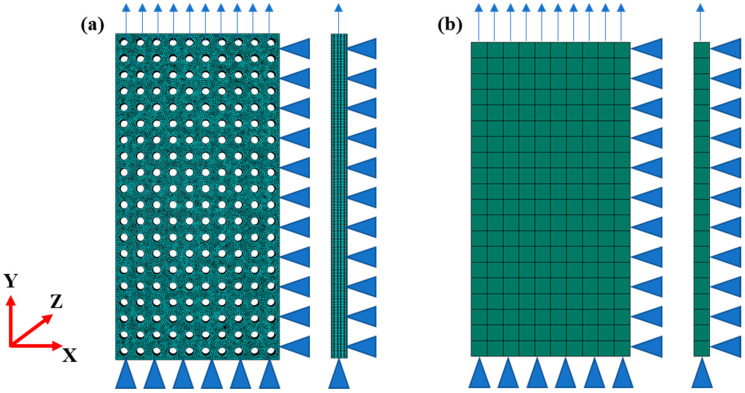
The loading conditions of finite element modeling: (**a**) the cellular material and (**b**) the equivalent material. The arrows represent the loadings, and the triangle symbolizes the normal constraint.

This study employs the structural static general analysis method in ABAQUS 6.14, using the geometric models presented in Figure 2 for the finite element modeling. The C3D8R element with a minimum size of 0.1 mm is utilized to simulate cellular materials, while the C3D8R element with a fixed size of 1 mm is used to simulate equivalent materials. Typic finite element models are depicted in Figure 3 and summarized in Table 1 according to their respective numbers of elements for both cellular and equivalent materials. The loading conditions are illustrated in Figure 3, where referent points are employed to apply loading and constraint conditions. Vertical surface constraints are set on surfaces along X, Y, and Z directions, and tensile loading is applied along the Y direction at a loading rate of 1 mm/min with maximum deformation limited to 10 mm.

### 3.3. The Constitutive Models and Their Material Parameters for the Cellular Materials and Their Equivalent Materials

In order to calculate the mechanical properties of the cellular materials and their equivalent materials, two constitutive models should be employed for the basis material and equivalent material of the cellular materials, respectively.

#### 3.3.1. The Constitutive Model and the Material Parameters for the Cellular Material

**Figure 4:**
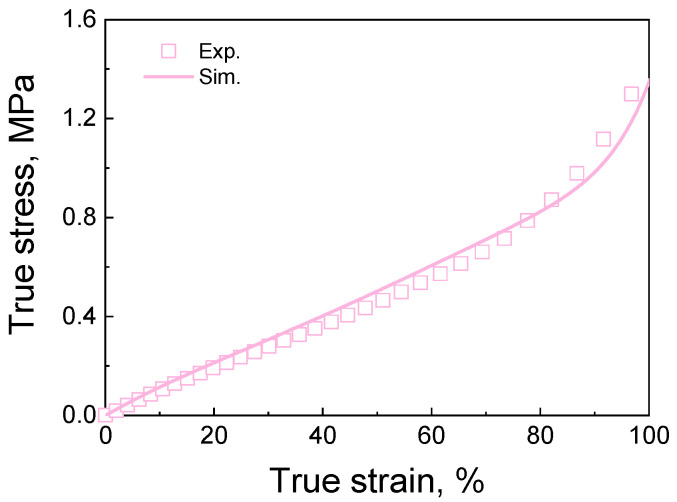
The true stress–strain curves of PDMS.

The PDMS material is simulated using the hyper-elastic Ogden model in the ABAQUS software [26]. The constitutive equations and responding parameter descriptions can be found in Appendix B. A cube with one C3D8R element is established to simulate the compression behavior. The parameters for the Ogden model are determined by fitting the experimental results, as shown in Table 2, and the experimental and simulated stress–strain curves are shown in Figure 4. The experiment details can be found in Appendix C. The hyper-elastic Ogden model and its parameters will be used to simulate the mechanical properties of cellular materials with different deformation features.

#### 3.3.2. The Constitutive Model and the Material Parameters for the Equivalent Material

**Figure 5:**
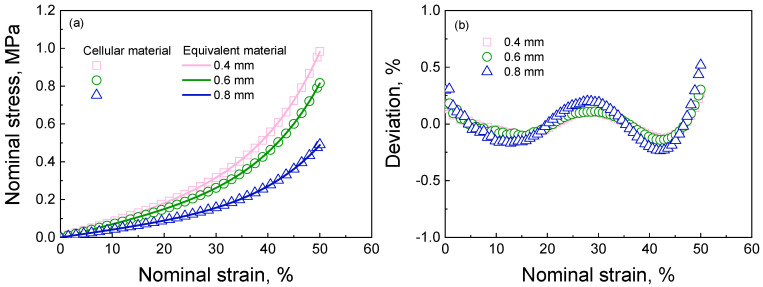
(**a**) The nominal stress−strain curves of uniformly distributed cellular materials and corresponding elements with different pore diameters; (**b**) the deviations between the results of the uniformly distributed cellular materials and their corresponding equivalent material. The deviations are computed using the equation (S_CM_ − S_EM_)/S_CM_ × 100. S_CM_ and S_EM_ denote the stress values of the cellular and equivalent materials at the same strain, respectively.

According to the sets in Section 3.1 and Section 3.2, the mechanical properties of three uniformly distributed cellular materials are computed using the hyper-elastic Ogden model in Section 3.3.1 and the parameters of the basis material in Table 2. The computed results for these uniformly cellular materials are shown in Figure 5a. It is observed that stress increases with a decrease in the pore diameter at the same strain. In addition, the computed effective Poisson’s ratio for the cellular materials with diameters of 0.8, 0.6, and 0.4 mm is 0.343, 0.298, and 0.187, respectively. The investigated cellular materials exhibit hyperelastic behaviors and compressibility characteristics due to the inherent hyperelasticity of the basis material and the presence of pores within the cellular materials. As the basic data points, these obtained results will serve as inputs for determining the mechanical properties of the equivalent material. Subsequently, the uniformly distributed cellular materials and their corresponding equivalent materials will be taken as examples.

According to the structure features of the cellular materials, a hyper-foam model [26] is employed to describe the deformation features of the equivalent materials. The details of the hyper-foam model can be found in Appendix D. By fitting the results of cellular materials in Figure 5a, the necessary parameters for this hyper-foam model can be determined (as shown in Table 3). Using the hyper-foam model and its parameters, the simulated stress–strain curves for equivalent materials closely match the results of cellular materials (as seen in Figure 5a). Similarly, the setups for finite element modeling are shown in Section 3.2. The nominal stress–strain curves of uniformly distributed cellular materials and their equivalent materials are shown in Figure 5a. The deviations between the cellular and equivalent materials’ results are computed, as shown in Figure 5b. It can be seen that the deviations range from −0.25% to 0.5%, thereby ensuring the validity of the proposed equivalent method. In addition, Figure 5 demonstrates a decrease in the stress responses with an increase in the pore diameter, indicating a reduction in the equivalent modulus associated with larger pore sizes. The obtained equivalent stress responses will be used to compute other equivalent materials with different deformation features in Section 4 using the hyper-foam model.

## 4. Results and Discussions

### 4.1. The Equivalent Computation Results for the Ordered and Random Cellular Materials

#### 4.1.1. The Deformation Response of the Cellular and Equivalent Materials with Different Deformation Features under Axial Tension

**Figure 6:**
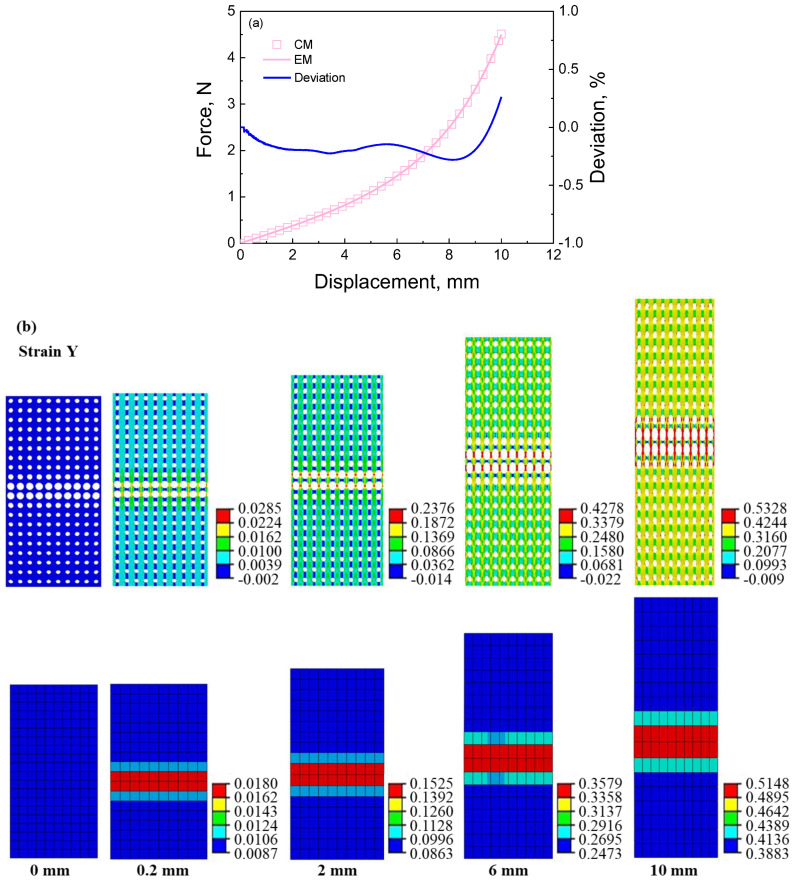

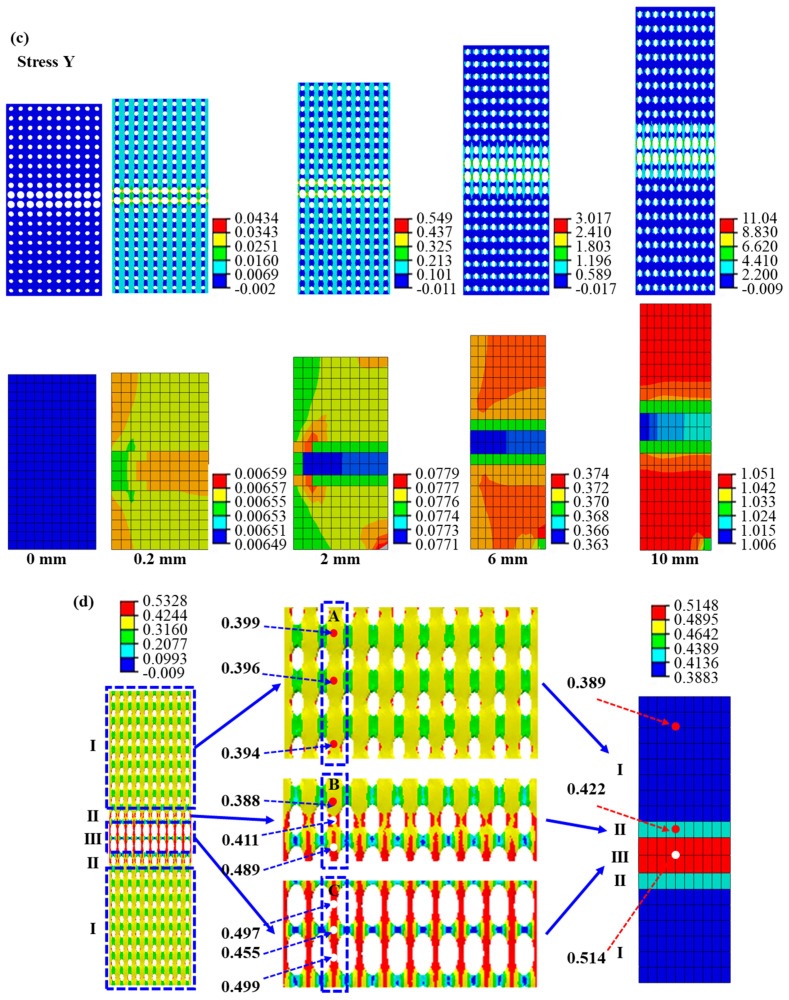

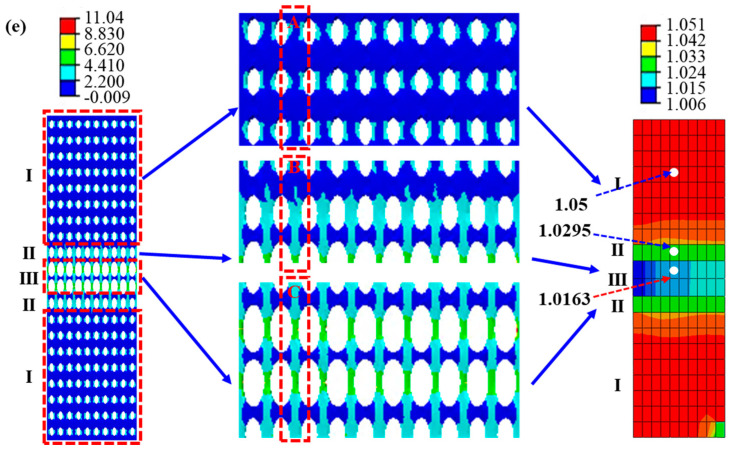
The results of cellular material and its corresponding equivalent material with stretching feature under tensile loading: (**a**) force–displacement curves and the deviation between the results of cellular material and equivalent material; (**b**) strain contour of cellular material and its corresponding equivalent material at different displacements during the tensile process; (**c**) stress contour of cellular material and its corresponding equivalent material at different displacements during the tensile process; (**d**) the zoom−in strain contour of cellular material and its corresponding equivalent material at displacement of 10 mm; (**e**) the zoom-in stress contour of cellular material and its corresponding equivalent material at displacement of 10 mm. CM denotes cellular material, and EM denotes equivalent material. In the figure, I, II, and III denote the numbers of their right regions. In (**b**,**c**), the first and second rows exhibit the contour of cellular material and its corresponding equivalent material from up to down, respectively, and the displacement increases from 0 mm to 10 mm from left to right. The original states at a displacement of 0 mm represent the no-stress/no-strain state. The same arrangement is shown in the contour image below.

**Figure 7:**
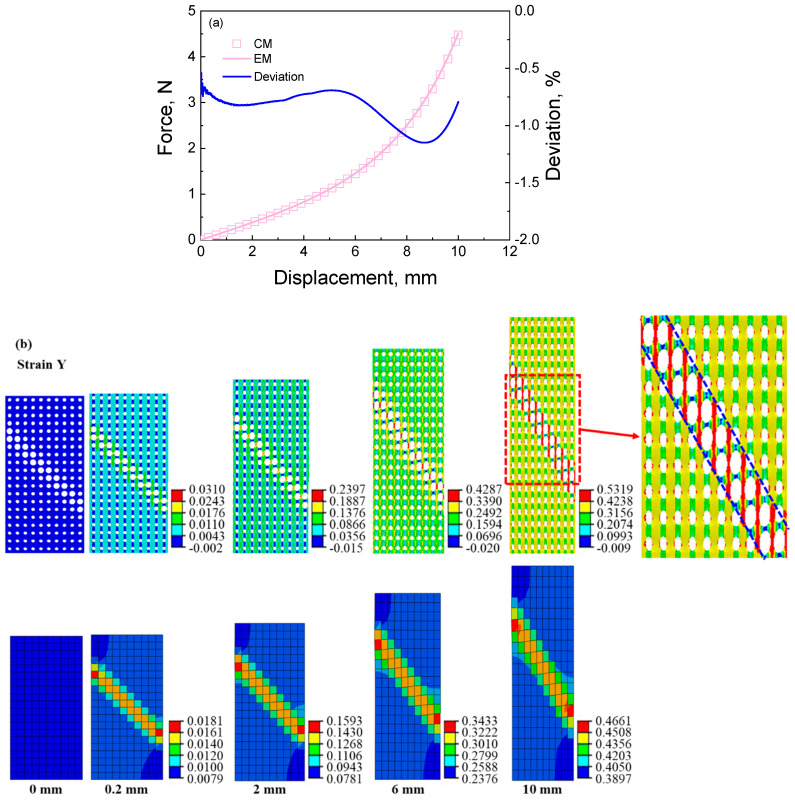

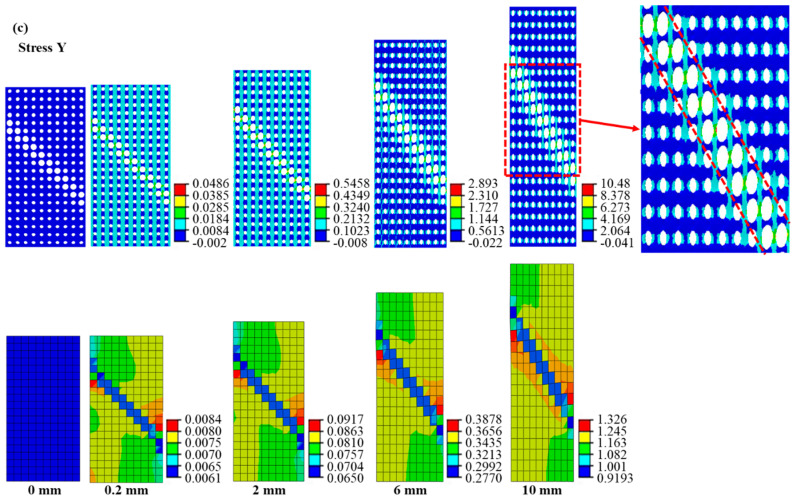
The results of cellular material and its corresponding equivalent material with shearing feature under tensile loading: (**a**) force–displacement curves and the deviation between the results of cellular material and equivalent material; (**b**) strain contour of cellular material and its corresponding equivalent material at different displacements during the tensile process; (**c**) stress contour of cellular material and its corresponding equivalent material at different displacements during the tensile process.

**Figure 8:**
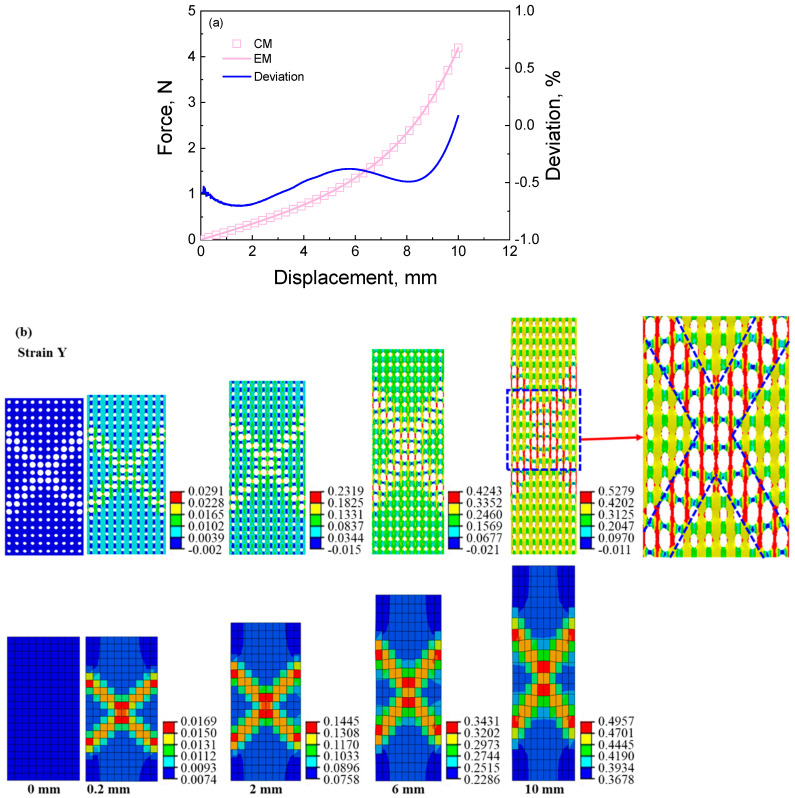

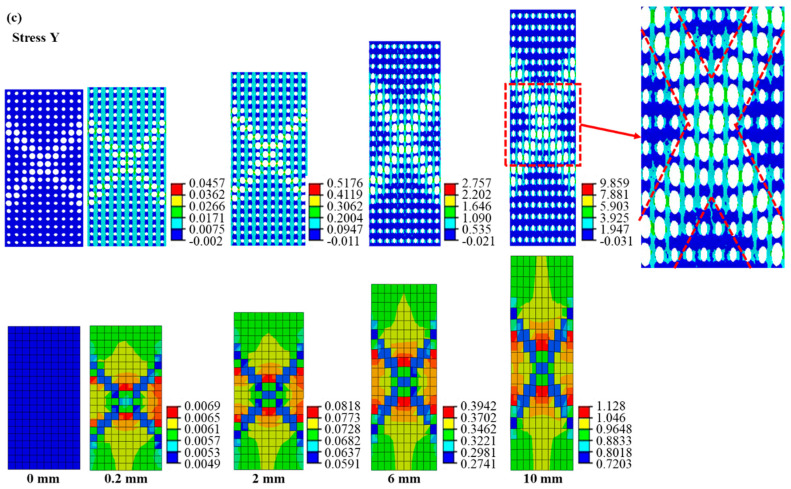
The results of cellular material and its corresponding equivalent material with double shearing feature under tensile loading: (**a**) force–displacement curves and the deviation between the results of cellular material and equivalent material; (**b**) strain contour of cellular material and its corresponding equivalent material at different displacements during the tensile process; (**c**) stress contour of cellular material and its corresponding equivalent material at different displacements during the tensile process.

**Figure 9:**
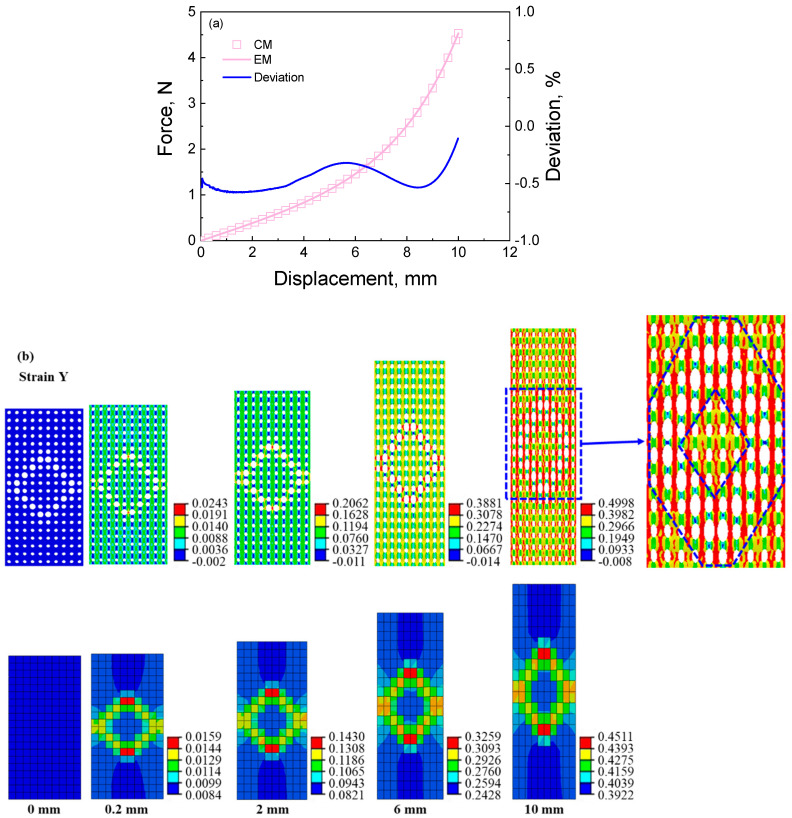

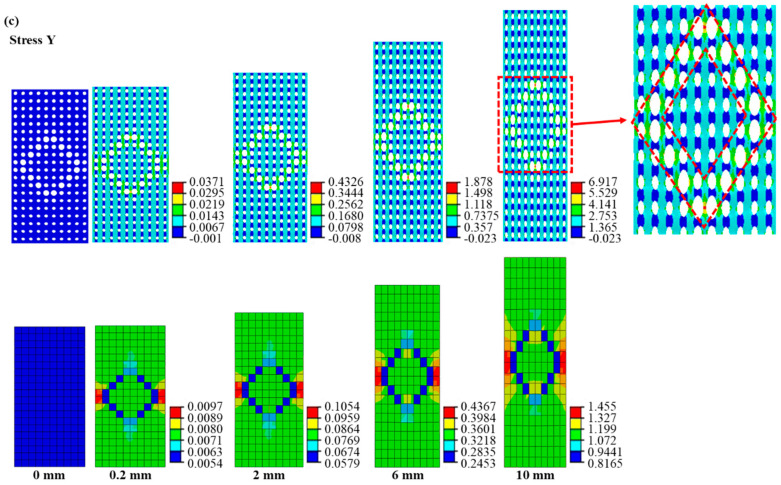
The results of cellular material and its corresponding equivalent material with circular deforming feature under tensile loading: (**a**) force–displacement curves and the deviation between the results of cellular material and equivalent material; (**b**) strain contour of cellular material and its corresponding equivalent material at different displacements during the tensile process; (**c**) stress contour of cellular material and its corresponding equivalent material at different displacements during the tensile process.

The calculated results for the different cellular materials and their equivalent materials under tensile loading are shown in Figure 6, Figure 7, Figure 8 and Figure 9. Initially, these results depict the force-displacement curves of the cellular materials and their equivalent materials. Subsequently, strain and stress contours are presented at various displacements to understand the deformation response across different deformation features. Following this, a comparative analysis is conducted between the contours of the cellular materials and their equivalent materials to clarify the equivalent principle.

From the force–displacement curves shown in Figure 6, Figure 7, Figure 8 and Figure 9, it can be seen that the calculated curves of equivalent materials agree with the results of cellular materials. The deviations between curves of the cellular materials (CM) and equivalent materials (EM) are computed as (CM − EM)/CM × 100, which should be as small as possible to ensure the validity of the equivalent calculation method. As shown in Figure 6, Figure 7, Figure 8 and Figure 9, all the deviations are less than 1.25%, indicating that this equivalent method can accurately describe the force–deformation responses of cellular materials. Furthermore, stress and strain contours at different deformation stages are extracted to analyze the deformation features, as shown in Figure 6b,c, Figure 7b,c, Figure 8b,c and Figure 9b,c.

Figure 6 presents the deformation responses of the cellular material with a stretching feature under tensile loading. The maximum diameter pores are predominantly located in the middle of the sample, resulting in a strain localization in the middle of the cellular material and its equivalent material, as shown in Figure 6b. Additionally, stress localization is also observed in the middle two rows of the samples, as shown in Figure 6c. However, there exists a disparity between the distributions of strain and stress values within this localization region. Specifically, the maximum strain appears in the middle two rows of the cellular material and its equivalent material; the maximum stress only appears in the middle two rows of the cellular material, but the minimum stress appears in them of the equivalent material. This discrepancy can be attributed to the distribution of the pore influencing the modulus characteristics since bigger pore diameters correspond to smaller equivalent moduli. Consequently, lower stresses are observed within the regions containing larger pore diameters in the middle part of the equivalent material. Despite some variations in the contours, the force–displacement curve of the equivalent material is predicted successfully using the equivalent calculation method.

In order to analyze the equivalent principles of the equivalent calculation method, we extract the details of the strain contour and stress contour at a displacement of 10 mm, as shown in Figure 6d,e. From Figure 6d,e, the sample is divided into three types of regions, including (I) the uniform deformation region at both ends, (II) the transitional deformation region, and (III) the localized deformation region. The zoom-in contours are extracted in the middle of the images. From Figure 6d, it can be seen that the strain localization appears in the region (III) of the cellular and equivalent materials. First, it is found that the pore wall in the region (I) along the loading direction appears yellow, with a strain range between 0.3160 and 0.4244. We extract three strain values in region A, such as 0.394, 0.396, and 0.399. Similarly, the strain value at an arbitrary point of the equivalent material is extracted as 0.389, which is approximate but slightly smaller than the strain value in region A of the cellular material. Then, the pore wall in region B along the loading direction appears from yellow to red, and its strain value changes from 0.3160 to 0.5328. We extract three strain values in region B, such as 0.388, 0.411, and 0.489. The strain value at an arbitrary point of the equivalent material is 0.422, approximating the average strain value in region B. Next, the pore wall in the region (III) along the loading direction appears red, and its strain value changes from 0.4244 to 0.5328. We extract three strain values in region C, such as 0.497, 0.455, and 0.499. The strain value at an arbitrary point of the equivalent material is 0.514, being in the strain range in region C. The above descriptions indicate that the strain distribution of the cellular material is predicted by the equivalent material model using the equivalent calculation method. The shortcoming is that the strain of the equivalent material in each region is uniform instead of non-uniform in the cellular material due to the influence of pores. It does not still affect the strain distribution with pore distribution.

Figure 6e also presents a stress localization in the cellular material, with the high-stress region in the middle of the sample. In the constant, the equivalent material presents a reversed stress localization, with a low-stress region in the middle of the sample. In order to clarify the detailed difference in stress distribution between cellular material and equivalent material, we analyze the stress values in different regions. For instance, from Figure 6e, the pore wall in the region (I) of the cellular material along the loading direction presents blue, with a stress range between 0 and 2.2 MPa; the pore wall in the region (II) along the loading direction presents from blue to blue-green, with a stress range between 0 and 4.41 MPa; the pore wall in the region (III) along the loading direction presents from blue-green to green, with a stress range between 4.41 and 6.62 MPa; the maximum stress reaches 11.04 MPa in the stress contour due to the stress concentration. The stress distribution of the cellular material indicates that the stress increases with porosity since the effective section area decreases with the increase in porosity under the same force. After that, as seen in the stress distribution of the equivalent material in Figure 6e, the stress (1.05 MPa) in the region (I) is larger than that (1.0295 MPa) in the region (II), and the stress (1.0295 MPa) in the region (II) is larger than that (1.0163 MPa) in the region (III). Since the equivalent material has a more uniform section and a larger section area than cellular material, it results in a smaller stress than the cellular material at the same force. At the same time, the stress distribution is reversed, as is the pore distribution. In addition, the stress in the equivalent material is always smaller than that in the cellular material, especially the maximum stress. It is because the equivalent material has a larger section area than cellular material. In addition, the stress concentration is easily produced in the cellular material’s deformation process. The above descriptions indicate that the stress distribution of the cellular material can also be reversely predicted by the equivalent material model using the equivalent calculation method. The larger the stress in the cellular material, the smaller the stress in the equivalent material.

The analyzed results of the deformation response of the cellular material with the stretching feature can be used to help understand the deformation response of the cellular material with other deformation features. From Figure 7b, Figure 8b and Figure 9b, it can be seen that the strain localizations appear in the region with deformation features. For instance, the strain localization of the equivalent material presents a shearing deformation feature since the big pore distributes with a “backward slash” shape, shown in Figure 7b. In the strain contour of the cellular material, a large strain appears along the big pore wall. As seen in Figure 7c, the stress distribution is related to the strain distribution. The maximum stress distribution region of the cellular material is the minimum stress distribution of the equivalent material since the equivalent modulus of the equivalent material is small in the shearing deformation feature region. The stress concentration of the cellular material appears in the strain concentration, but the small stress distribution of the equivalent material appears in the strain concentration.

The same situation appears in the stress and strain distributions of the cellular material with the double stretching and circular deforming features. Their stress and strain distributions present the “X” shape and “loop” shape deformation features, as shown in Figure 8 and Figure 9. The strain and its distribution of the cellular material are reproduced by calculating the equivalent material and are approximated to those of the equivalent material. The computed results indicate that the strain distribution of the cellular material is predicted by the equivalent material model using the equivalent calculation method. The stress value of the equivalent material is also slightly smaller than that of the cellular material, and the stress distribution of the cellular material is also reversely predicted by the equivalent material model using the equivalent calculation method.

Nevertheless, the above results indicate that the equivalent method can describe the deformation mode of cellular materials with different deformation features under tensile loading. Although there is some difference in the value, the distribution of stress and strain in cellular materials is still simulated using the equivalent method. The method mentioned above further proves the effectiveness of the equivalent method.

#### 4.1.2. The Deformation Response of the Cellular and Equivalent Materials under Shearing Loading

**Figure 10:**
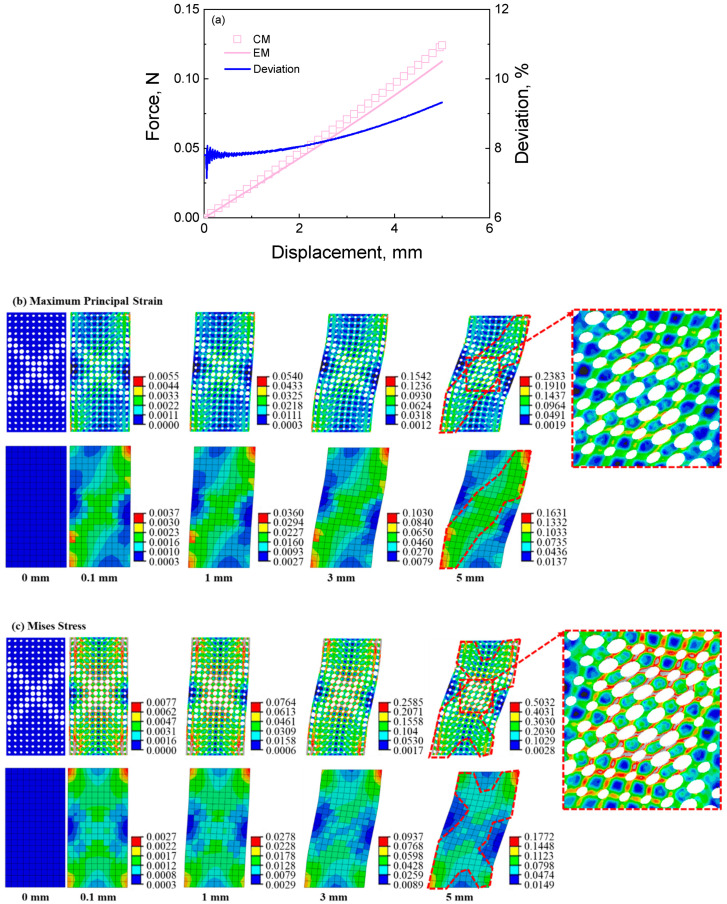
The results of cellular material and its corresponding equivalent material with shearing feature under shearing loading: (**a**) force-displacement curves and the deviation between the results of cellular material and equivalent material; (**b**) strain contour of cellular material and its corresponding equivalent material at different displacements during the tensile process; (**c**) stress contour of cellular material and its corresponding equivalent material at different displacements during the tensile process.

The calculated results of cellular material and its corresponding equivalent material with the double shearing feature under shearing loading are shown in Figure 10. The maximum loading displacement is 5 mm, and the loading rate is 1 mm/min. It can be seen from Figure 10a that the simulated force-displacement curve is approximately linear. The average deviation between the results of cellular material and equivalent material under shearing loading is about 8.316%, which is slightly bigger than that under tensile loading. From Figure 10b,c, the equivalent method captures the deformation mode of cellular material predicted by the direct numerical simulation. The stress and strain contours present a shearing deformation mode. In addition, the equivalent method captures the distributions of strain and stress in the cellular material predicted by the direct numerical simulation.

The red dashed line in Figure 10b shows that the same shapes of strain distribution are obtained in the results of the cellular material and its corresponding equivalent material. From the strain contour, the maximum strain of the cellular material is larger than that of the equivalent material, which is more remarkable than the deformation response under tensile loading. From the zoom-in image in Figure 10b, the pore presents shearing deformation with a non-uniform strain distribution, and the maximum strain distributes in the pore wall along the shearing deformation direction. It differs from the pore wall’s axial deformation under tensile loading, as shown in Figure 8b. The above-mentioned may be the reason why the average deviation between the results of cellular material and equivalent material under shearing loading is bigger than that under tensile loading.

In Figure 10c, the high stress of cellular material is distributed in the pore wall with a large diameter; in contrast, the low stress of equivalent material is distributed in the low modulus region, like an italic “*X*” shape. The phenomenon is in line with the results under tensile loading. In addition, the pore walls present shearing deformation under the shearing loading. The stress distribution of cellular materials is also reversely predicted by the equivalent calculation method. The above results indicate that the equivalent method can describe the deformation response of cellular materials under shearing loading.

#### 4.1.3. The Prediction for the Deformation Response of the Random Cellular Material under Axial Tension

**Figure 11:**
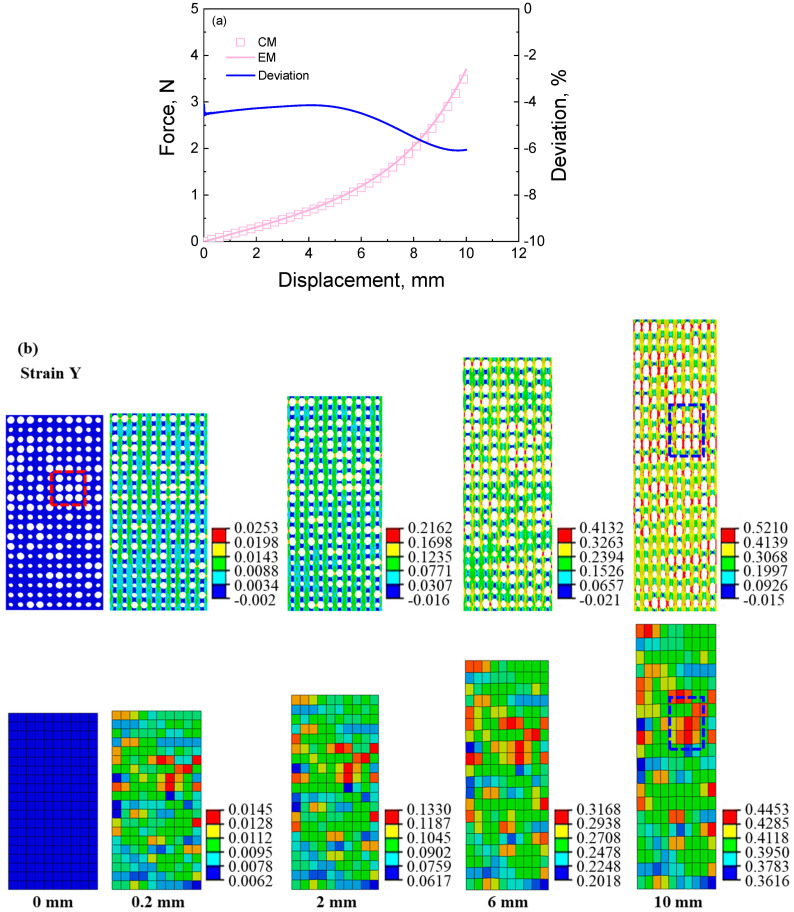

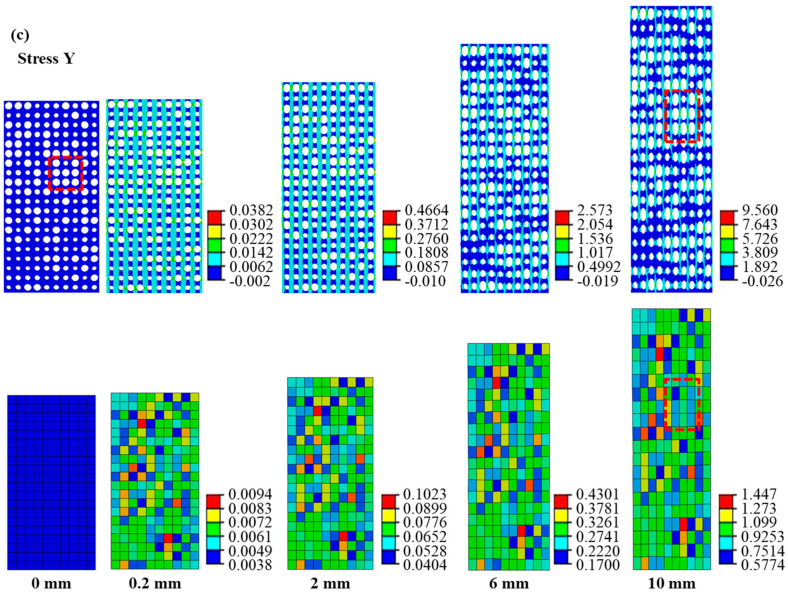
The simulated results of the random cellular material under tensile loading: (**a**) force–displacement curves and the deviation between the results of cellular material and equivalent material; (**b**) strain contour of cellular material and its corresponding equivalent material at different displacements during the tensile process; (**c**) stress contour of cellular material and its corresponding equivalent material at different displacements during the tensile process.

The simulated results for the random cellular material under axial tension are shown in Figure 11. From Figure 11a, the simulated force–displacement curve of equivalent materials agrees with the result of the cellular material with an average deviation of 4.74%. From Figure 11b,c, it can be seen that the equivalent method can capture the strain and stress distributions under tensile loading. The deformation region concentrates on the big pores in the cellular material and the low-modulus elements in the equivalent material. On the contrary, the high stress is distributed on the wall of big pores in the cellular material, and the low stress is distributed on the element with a low modulus in the cellular material. In addition, the stress and strain distributions of the random cellular material and its corresponding equivalent material are not completely in line with the big pore distribution, as seen from the dashed box in Figure 11b,c. It may be because the surrounding structures affect the deformation feature. It is still a complex question that will continue to be investigated. Nevertheless, the above results indicate that the equivalent method can describe the deformation behavior of the random cellular material under tensile loading.

#### 4.1.4. The Effect of the Randomness on the Deformation Response of the Random Cellular Material under Axial Tension

**Figure 12:**
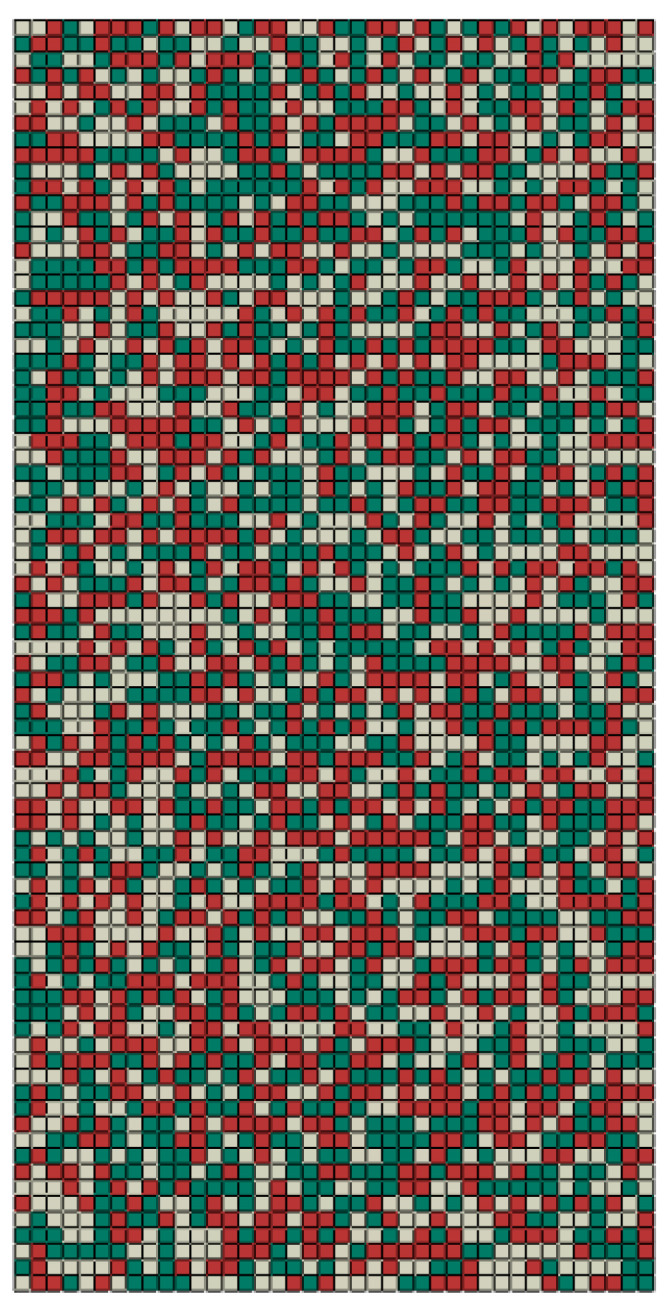
The model of an equivalent material with 6400 (40 × 80 × 2) elements for calculating the deformation behaviors of a random cellular material. The green, white, and red codes denote the equivalent cells with pore diameters of 0.4, 0.6, and 0.8 mm, respectively.

In order to further verify the equivalent method and its computational efficiency and accuracy, a random equivalent material with 6400 elements is computed under tensile loading, as shown in Figure 12. The 6400 elements represent 6400 cells in the cellular material, indicating the high computation burden.

**Figure 13:**
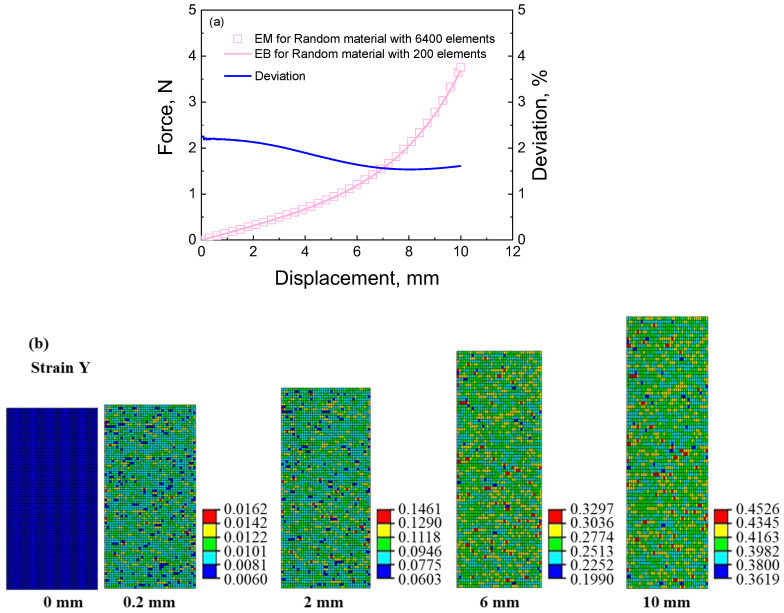

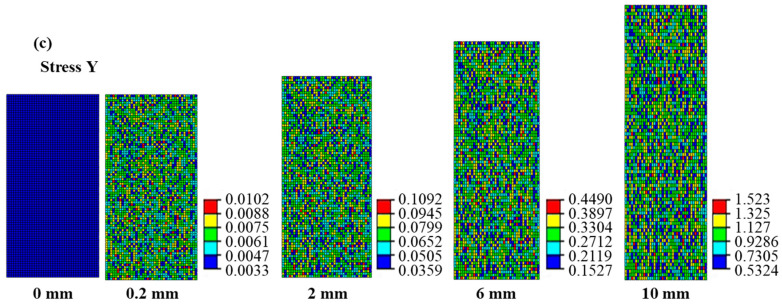
The simulated results of the equivalent material for calculating the deformation behaviors of the random cellular material: (**a**) force–displacement curves and the deviation between the equivalent material with 6400 elements and that with 200 elements; (**b**) strain contour of random equivalent material at different displacements during the tensile process; (**c**) stress contour of random equivalent material at different displacements during the tensile process.

Like the random model with 200 elements in Figure 11, the random equivalent material model with 6400 elements is built by combining the finite element software and Python scripts, as shown in Appendix A. The ratio of the three types of unit cells in the random equivalent material with 6400 elements is the same as that with 200 elements. The randomness increases with the number of unit cells. The loading conditions and material parameters are the same as those in Section 3. The simulated results are shown in Figure 13. From Figure 13a, it can be obtained that the average deviation between the force–displacement curve of the random model with 200 elements and that with 6400 elements is 1.819%. It indicates that the equivalent method can reasonably describe their mechanical properties. Also, it infers that the influence of the random distribution of unit cells on the macro mechanical properties can be neglected under the same unit cells. However, the size of the micro-structure can affect the macro mechanical properties [14,27]. From Figure 13b,c, the deformation model presents uniform deformation macroscopically and non-uniform deformation microscopically. Compared with the stress and strain distributions in Figure 11b,c, the greater the number of elements, the stronger the randomness is, and the better the uniformity.

### 4.2. The Computational Efficiency between the Cellular and Their Equivalent Materials

The computational efficiency under tension loading is concluded in Table 1 to clarify the advantage of the equivalent method. The number of elements, the size of the calculated results, and the computing time of the job are discussed. It can be seen from Table 1 that, compared with the cellular material, the number of elements in the equivalent materials is averagely reduced by 99.78%, the size of calculated results in the equivalent material is averagely reduced by 99.62%, and the computing time in the equivalent material is averagely reduced by 99.78%. The above results indicate that the computing efficiency of the equivalent method has been greatly improved. Comparing the results of cellular materials and equivalent materials, the average deviation for cellular materials with a uniform distribution is 0.002%, which approaches zero. The average deviation for cellular materials with other deformation features is 0.492% under tensile loading and 8.32% under shearing loading. These low deviations also indicate that the equivalent method can improve the computational efficiency of cellular materials.

In finite element modeling, the selection of the constitutive model is key. Based on the hyper-elastic PDMS and its cellular material, this method selects the hyper-elastic and hyper-foam models to simulate the mechanical properties of the PDMS and the equivalent material of the cellular material. Whether this method suits the mechanical properties of other materials, such as viscoelastic material, elastic-plastic material, visco-plastic material, and so on, still needs further verification. Additionally, the anisotropic responses in cellular materials may be a difficult problem for applications and should be considered further in the constitutive model. For the different cells, the mechanical properties are different. Completing equivalence through the material parameters is still a key and a difficulty. In this study, the pore and topology of the unit cell are regular, and the equivalent method is easy to operate. However, in the equivalent calculation of the irregular cellular material, the randomness of the pore, including the pore diameter and distribution, could increase the difficulty of determining the RVE. In general, this proposed equivalent approach achieves the aim of equivalently calculating. By employing the equivalent approach in practical applications, we first predict the macroscopic mechanical behaviors and obtain the total force–displacement curves. After obtaining a holistic understanding, we examine the local deformation in detail, paying special attention to regions that exhibit significant strain distributions and also assessing the correction between the maximum and ultimate strains of the basis material. The results of these calculations can serve as a foundation for prompt decision-making since they are characterized by high efficiency. If more intricate calculations and detailed results are desired, localized, meticulous computations become necessary. Overall, this approach conveniently provides preliminary assessments for engineering applications.

## 5. Conclusions

This study proposes a novel and straightforward equivalent method for computing the mechanical properties of random and ordered hyper-elastic cellular materials with regular unit cells. This equivalent method is realized by integrating the finite element method with the distribution characteristic of pores. The efficacy of the proposed method is verified by comparing the simulated results of cellular materials with those of equivalent materials. The main conclusions are obtained as follows:

(1) The equivalent calculation method can reasonably compute the macroscopic force-displacement curves of the random and ordered cellular materials with different deformation features. The average deviation is 1.67% when contrasting the results of the cellular materials and equivalent materials. The deviation is more pronounced in random cellular materials than ordered cellular materials due to intricate deformations arising from neighboring pores. Shearing loading produces a more noticeable deviation than tensile loading owing to the complex shearing deformation endured by pore walls.
(2) The equivalent calculation method can reasonably describe the stress and strain distributions. Equivalent materials exhibit localized strains in regions with distinctive deformation features, with cellular material strains closely approximating their equivalent counterparts. Moreover, the equivalent material model can predict the cellular material’s stress distribution in reverse. Notably, the region of maximum stress distribution in cellular material corresponds to the minimum stress distribution in the equivalent material, a result of the smaller equivalent modulus within the deformation feature zone. Discrepancies between cellular and equivalent material contours stem from surrounding structural influences.
(3) The equivalent calculation method saves huge computational burdens. Compared with the results of the cellular materials, equivalent materials showcase a substantial reduction, presenting 99.78% in element count, 99.62% in result size, and 99.78% in computation time.

The equivalent method will be expected to be applied to the pre-simulation of the prototype, providing a reference for the final configuration of the structure.

## Figures and Tables

**Table 1:** The computational efficiency of the equivalent method.

Sample	Number of Elements			The Size of Computational Results (Mega Byte)			Computing Time (s)			Average Deviation
	CM	EM	ICE (%)	CM	EM	ICE (%)	CM	EM	ICE (%)	
Uniform deforming feature with a diameter of 0.4 mm	103,010	200	99.81	6492.16	22	99.66	8718	17.5	99.80	0.0035%
Uniform deforming feature with a diameter of 0.6 mm	97,845	200	99.80	6215.68	22	99.65	8274.7	17.6	99.79	0.0024%
Uniform deforming feature with a diameter of 0.8 mm	64,465	200	99.69	4229.12	22	99.48	4735.3	17.4	99.63	0.00015%
Stretching feature	99,630	200	99.80	6266.88	22	99.65	8534	17.2	99.80	0.170%
Shearing feature	93,105	200	99.76	5898.24	22	99.63	8076	16.9	99.79	0.851%
Double shearing feature	94,740	200	99.79	6010.88	22	99.63	8153	16.9	99.79	0.489%
Circular deforming feature	98,725	200	99.80	6236.16	22	99.65	8423	17.7	99.79	0.458%
Double shearing feature under shearing loading	94,740	200	99.79	6010.88	22.1	99.63	10,902	17.2	99.84	8.316%
Random deforming feature	88,540	200	99.77	5601.28	22	99.61	10,166	17.3	99.83	4.74%

Mark: CM denotes the cellular material, EM denotes the equivalent material, and ICE denotes the increment in the computational efficiency, which equals (CM − EM)/CM × 100.

**Table 2:** Parameters of the hyper-elastic Ogden model for PDMS.

*μ*_1_/MPa	*μ*_2_/MPa	*μ*_3_/MPa	*a*_1_	*a*_2_	*a*_3_	*K*/MPa
0.43	0.011	−0.015	4.55	16.54	−7.22	332.72

**Table 3:** Parameters of the hyper-foam model for equivalent material.

	*μ*_1_/MPa	*μ*_2_/MPa	*μ*_3_/MPa	*a*_1_	*a*_2_	*a*_3_	*β*_1_/MPa
①	−1.244	1.249	0.130	14.448	14.454	4.866	−980.116
②	−2.148	1.945	0431	10.762	10.994	6.914	−110.087
③	−2.577	2.334	0.519	10.772	11.004	6.923	−57.183

Remark: ①, ②, and ③ denote the parameters for the equivalent materials with diameters of 0.8, 0.6, and 0.4 mm, respectively.

**Figure A1:**
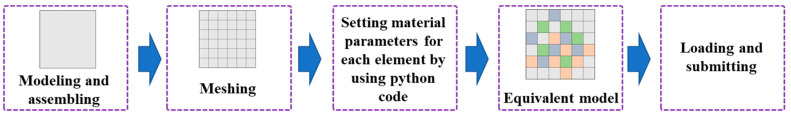
The main process of finite element modeling.

**Figure A2:**
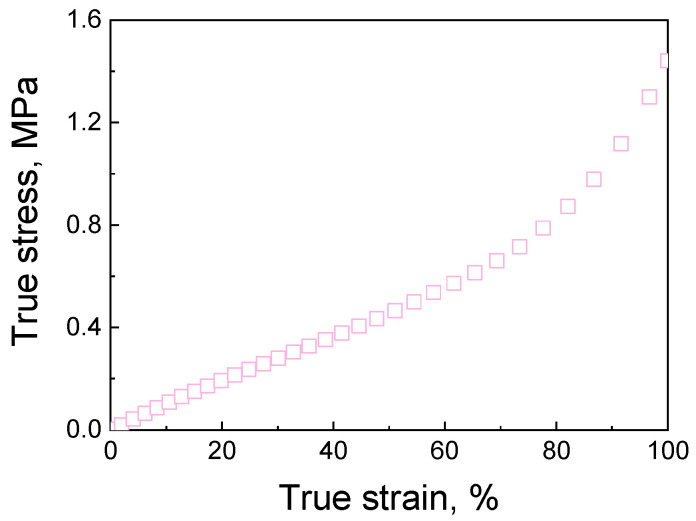
The experimental true stress-strain curves of PDMS.

## Data Availability

Not applicable.

## Appendix A. Python Script for Establishing the Random Equivalent Material

In order to establish the random equivalent material, the randomness can be realized using a Python script. The main processes of finite element modeling using a Python script are shown in Figure A1. First, establish the model in the ABAQUS and then mesh it. Next, set the material parameters for each element using a Python script. An equivalent model with different material parameters is obtained. Finally, the loading conditions are applied to the model. The corresponding Python script is given as follows:

We only set material parameters for each element using a Python script, and other operations are completed in the finite element software ABAQUS. The script fits the random materials with 200 elements and 6400 elements.

The Python script is given as follows:

from abaqus import *
        from abaqus Constants import *
        import random
        myModel = mdb.models[“Model-random”]
        myPart = myModel.parts[‘Part-1’]
        myElement = myPart.elements
        for iter in range(len(myElement)):
              randNum = random.randint(1, 3)
              setName = ‘Set-{0}’.format(iter)
              myPart.Set(elements=myElement[iter:iter+1], name=setName)
              myPart.SectionAssignment(region=myPart.sets[setName],
        sectionName=‘Section-{0}’.format(randNum), offset=0.0, 
              offsetType=MIDDLE_SURFACE, offsetField=”,
              thicknessAssignment=FROM_SECTION)
		

## Appendix B. The Hyperelastic Ogden Model

The energy potential of the Ogden model is as follows:

(A1) $$U=\sum_{i=1}^{N}\frac{2\mu_{i}}{\alpha_{i}^{2}}\left(\lambda_{1}^{\alpha_{i}}+\lambda_{2}^{\alpha_{i}}+\lambda_{3}^{\alpha_{i}}-3\right)+\frac{1}{2}K{\left(J-1\right)}^{2}$$

where $\mu_{i}$ and $\alpha_{i}$ denote the material parameters, K is the bulk modulus, J is volumetric strain, and $\lambda_{1}$, $\lambda_{2}$, $\lambda_{3}$ denote the principal stretch ratio.

The stress-strain relations can be derived as follows:

(A2) $$\sigma_{k}=\frac{2}{J}\sum_{i=1}^{N}\frac{\mu_{i}}{\alpha_{i}}\left[\lambda_{k}^{\alpha_{i}}-\frac{1}{3}\left(\lambda_{1}^{\alpha_{i}}+\lambda_{2}^{\alpha_{i}}+\lambda_{3}^{\alpha_{i}}\right)\right]+K\left(J-1\right)$$

## Appendix C. Experimental Method and Results

This study uses a hyper-elastic polydimethylsiloxane (PDMS) as the basis material for the cellular materials. In order to obtain the basic material parameter for the finite element modeling, a specimen with a diameter of 10 mm and a height of 10 mm is used to conduct the axial compression experiment with a loading rate of 1 mm/min. A material testing machine with a maximum load of 1 kN was used. The experimental true stress-strain curve is given in Figure A2.

## Appendix D. The Hyper-Foam Model

The strain energy function of the hyper-foam model is as follows:

(A3) $$U=\sum_{i=1}^{N}\frac{2\mu_{i}}{\alpha_{i}^{2}}\left[\left(\lambda_{1}^{\alpha_{i}}+\lambda_{2}^{\alpha_{i}}+\lambda_{3}^{\alpha_{i}}-3\right)+\frac{1}{\beta_{i}}\left(J_{}^{-\alpha_{i}\beta_{i}}-1\right)\right]$$

where $\mu_{i}$, $\alpha_{i}$, and $\beta_{i}$ denote the material constants, J is volumetric strain ratio, and $\lambda_{1}$, $\lambda_{2}$, $\lambda_{3}$ denote the principal stretches.

The stress-strain relations can be derived as follows:

(A4) $$T_{k}=\frac{\partial U}{\partial \lambda_{k}}=\frac{2}{\lambda_{k}}\sum_{i=1}^{N}\frac{\mu_{i}}{\alpha_{i}}\left[\lambda_{k}^{\alpha_{i}}-J_{}^{-\alpha_{i}\beta_{i}}\right]$$
